# Cell Patterning for Liver Tissue Engineering via Dielectrophoretic Mechanisms

**DOI:** 10.3390/s140711714

**Published:** 2014-07-02

**Authors:** Wan Nurlina Wan Yahya, Nahrizul Adib Kadri, Fatimah Ibrahim

**Affiliations:** 1 Department of Biomedical Engineering, Faculty of Engineering, University of Malaya, Kuala Lumpur 50603, Malaysia; E-Mails: wnurlina2@siswa.um.edu.my (W.N.W.Y.); fatimah@um.edu.my (F.I.); 2 Centre for Innovation in Medical Engineering (CIME), Faculty of Engineering, University of Malaya, Kuala Lumpur 50603, Malaysia

**Keywords:** cell patterning, dielectrophoresis, tissue engineering, hepatocytes, liver, artificial organ, artificial liver, engineered liver, lab-on-a-chip, BioMEMS liver

## Abstract

Liver transplantation is the most common treatment for patients with end-stage liver failure. However, liver transplantation is greatly limited by a shortage of donors. Liver tissue engineering may offer an alternative by providing an implantable engineered liver. Currently, diverse types of engineering approaches for *in vitro* liver cell culture are available, including scaffold-based methods, microfluidic platforms, and micropatterning techniques. Active cell patterning via dielectrophoretic (DEP) force showed some advantages over other methods, including high speed, ease of handling, high precision and being label-free. This article summarizes liver function and regenerative mechanisms for better understanding in developing engineered liver. We then review recent advances in liver tissue engineering techniques and focus on DEP-based cell patterning, including microelectrode design and patterning configuration.

## 1. Introduction

Every year, liver transplantation for end-stage liver failure becomes a highly critical issue due to the limited number of organ donors. As of 2013, more than 100,000 patients in United States were still on the waiting list, while the number of organ donors is less than half the number of needed organs [1]. In addition, the need for continuous immunosuppressive treatment for the organ recipient due to immune response issues has encouraged researchers to seek alternatives [2]. Hence, research in tissue engineering, an interdisciplinary field, has explored the possibilities of offering new solutions for organ failure or tissue loss [3].

Principally, cell culture technique is the key factor in tissue engineering in efforts to mimic the complex *in vivo* microenvironment. *In vivo*, hepatocyte cells are organized in a highly complex architecture with well interactions between non-parenchymal cells and the extracellular matrix (ECM) allowing hepatocytes to maintain their physiological duty. With standard cell culture methods, hepatocyte cells were cultured in planar Petri dishes, where continuous manual changes of media are needed. This static 2D cell culture system, however, shows poor cell viability, and the cells simply lose their phenotype function [4]. Thus, inspired by the biological structure and microenvironment of liver, diverse engineering approaches have been developed with certain features to precisely control the cellular microenvironment to permit better control of cellular behavior [5–8].

Herein, this article briefly describes liver functions and the remarkable regenerative mechanisms for additional understanding in developing an engineered liver. Next, a review on liver tissue engineering is presented, describing diverse techniques including scaffold-based approaches, cell sheet technology, microfluidic platforms and micropatterning. Realizing the importance of cell patterning in *in vitro* liver construction, one of the most popular electrical approaches for precisely manipulating cells into specific patterns, called dielectrophoresis (DEP), is given special attention here. We then review recent work on liver tissue engineering via dielectrophoretic mechanisms, the principal technologies and the key parameters needed for better patterning. In addition to application organ transplantation, the tissue construct also can be utilized for drug screening and biological studies with approachable integrated systems.

## 2. Liver

### 2.1. Liver Functions and Regenerative Mechanisms

The liver is a vital complex internal organ that plays a major part in the living body. This extraordinary organ is responsible for controlling body metabolism by chemically converting nutrients into energy; it also synthesizes substances needed by cells, such as carbohydrates [9], proteins [10] and fats [11]. To continuously sustain the living state of the body tissue, the liver also acts as a filter, detoxifying the undesired elements found in the blood and lymph circulatory system such as toxins and excess hormones [12]. Research has found that significant changes in liver function, such as immune dysfunction and chronic diseases such as cancer and fibromyalgia, can cause liver damage [13].

The normally functioning liver has a unique feature whereby the hepatocytes rarely proliferate in normal conditions but are able to regenerate upon the loss of hepatic tissue mass. Nearly a century ago, Higgins and Anderson showed the ability of the liver of the white rat to self-regenerate after performing a 70% partial hepatectomy, and the restoration was completed within just 3 weeks [14]. This finding has encouraged scientists to deepen the understanding of this remarkable event. Rhim *et al.* developed a transgenic mouse system to evaluate the regenerative capacity of hepatocytes. In their study, they showed that the transplantation of xenogeneic liver cells into albumin-urokinase (Alb-uPA) transgenic mice completely regenerated in several weeks with liver mass similar to that of the control. Moreover, adequate liver function was identified in the transplanted rat hepatocytes by secretion of proteins as well as drug metabolism and detoxification [15]. On the other hand, decreases in liver mass occurred when the functional capacity of the liver went beyond the body's ideal requirements.

**Figure 1.**
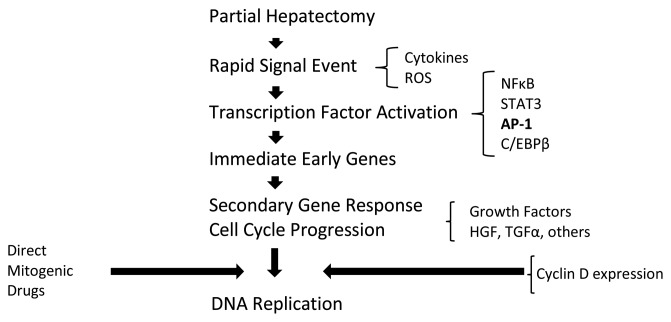
A broad outline of important events in liver generation. Reproduced from [16] with permission.

Extensive studies have been conducted to analyze the mechanisms that regulate the regenerative development using animal models, commonly mice, subjected to partial hepatectomy. Basically, hepatocytes regenerate in response to a series of various gene activations, growth factor production, and morphologic arrangement throughout several phases, as shown by the general flow in Figure 1 [16]. Every growth factor plays a definite role during the regenerative process, including hepatocyte growth factor (HGF), epidermal growth factor (EGF), transforming growth factor-α (TGF-α), tumor necrosis factor-α (TNF-α), interleukin-6 (IL-6), insulin and norepinephrine. However, dysregulation of these growth factors may lead to hepatocarcinogenesis [17]. Studies suggest that overexpression or imbalance of either growth stimulatory or inhibitory factors is fundamental in tumor development. Therefore, it is essential to deliberate these biological circumstances during tissue construction.

### 2.2. Liver Tissue Engineering

*In vivo*, liver cells live in a comfortable microenvironment in which adequate nutrients, growth factors and oxygen are supplied by the circulatory system and provide biochemical and mechanical interactions with the neighboring environment. Cells regularly receive numerous cues through communications between cells and the extracellular matrix (ECM) promoting differentiation, proliferation, growth and assembly to form a functional tissue. ECM components include collagen, laminin and fibronectin, which have proven to be favorable in hepatic development and regeneration in a variety of ways, such as networking with cell surface receptors and delivering cytokines [18,19]. Furthermore, control interactions between parenchymal and non-parenchymal cells are significant to preserve hepatocyte morphology and a variety of functions such as metabolism, detoxification [20] and protein synthesis [21], as described in Section 2.1.

Due to those resourceful surroundings, when enzymatically isolated hepatocytes are cultured in static and monolayer systems, they rapidly lose their morphology and many phenotypic functions. As discussed before, research has shown that liver cells have a high capacity to repair themselves. However, this remarkable ability is difficult to implement *in vitro*, but it is possible with the support of a suitable microenvironment prior to implantation. In consequence, by utilizing the principles of biology and engineering, functional engineered liver tissue could be developed to resemble the biological tissue by *in vitro* culture. This interdisciplinary field, called tissue engineering, offers a great opportunity to overcome the health issues regarding loss or damage of liver, drug toxicity and can be used to investigate deep within the liver's biological system.

#### 2.2.1. Engineering Approaches for *in vitro* Liver Cell Culture

Continued advancements in tissue engineering have provided appropriate environments on the micro scale to suit the micro dimensions of cells. Emerging microelectromechanical system (MEMS) technologies allow new opportunities to understand the electrochemical and mechanical processes responsible for changes in cell culture performance [22].

**Figure 2.**
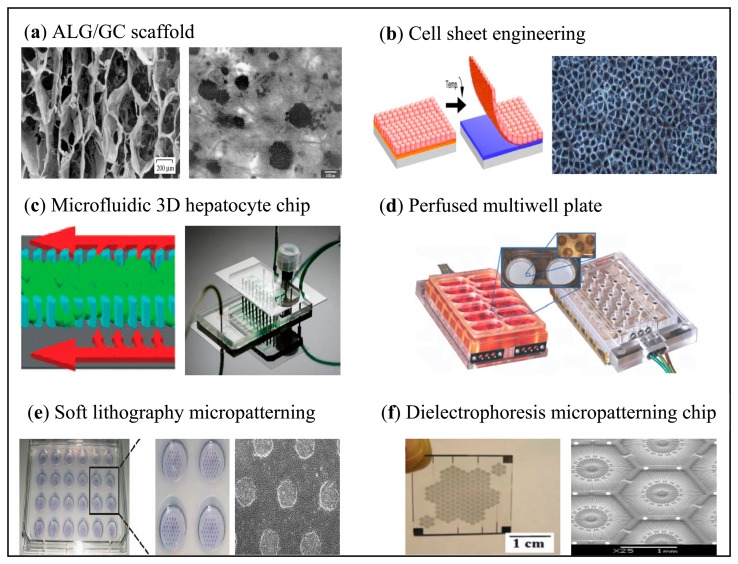
(**a**) SEM image of ALG/GC scaffold for hepatocytes attachment [23]. (**b**) Cell sheet technology for passive cell patterning using PIPAAm-grafted surface [24]. (**c**) Microfluidic 3D hepatocyte chip utilizing micro-pillars for cell culture [25]. (**d**) Perfused multi-well plate with an array of 12 scaffold-based bioreactors [26]. (**e**) Soft lithography to fabricate hepatocytes micropatterning in a multiwell format [27]. (**f**) Microelectrode for active liver cell patterning via DEP mechanism [28]. Reproduced with permission.

Unlike conventional apparatus, with the aim towards liver-on-a-chip, many recent tools have been developed with the ability to operate small volumes of fluid, are portable and easy to integrate with other systems, and are low-cost products for the purpose of commercialization. Figure 2 shows some techniques available for liver tissue engineering.

However, the major attraction of those tools for cell culture applications is the competency to imitate the *in vivo* microenvironment of cells with good cell-cell and cell-ECM interactions, optimum oxygen and nutrient supplies, precisely controlled temperature and pH, biochemical and mechanical stress and many other factors. In this section, we briefly review some engineering approaches that address some desirable parameters for generating liver tissue cultured *in vitro*. Moreover, a summary of representative literature of comparison between available engineering approaches specifically for liver tissue engineering is included in Table 1.

##### Scaffold-Based

Scaffolds are designed to function as *in vitro* ECM for cell culture to promote cell differentiation, proliferation and migration, and they gradually degrade upon implantation in the patient and are substituted by neo-tissue. Generally, scaffold structure should have a highly interconnected porous network to allow perfusion of gases, nutrients and growth factors to the cells; be three-dimensional (3D); be biodegradable, for easy elimination out of the body; be biocompatible with the host tissue; and possess good mechanical properties to support and sustain the preferred shape [29,31]. These structures can be fabricated from a wide variety of materials, either natural biomaterials or synthetic polymers.

One of the most widely used natural biomaterials for liver tissue engineering is alginate. In addition to its biocompatibility and low toxicity, the hydrophilic nature of alginate scaffold facilitates the efficient seeding of hepatocytes onto the sponge-like scaffold. Glicklis *et al.* observed the aggregation behavior of freshly isolated adult rat hepatocytes seeded within a 3D alginate-based scaffold [30]. This work showed that within 24 h after cell seeding, small groups of hepatocytes begin to appear, and by day 4, they become as large as the pore size of the scaffold and form spheroids with the presence of fibronectin. Within a week, the cells performed typical hepatocyte functions, such as secreting albumin and urea at the maximal rate, indicating that the alginate scaffold facilitated their functional expression. Unfortunately, poor mechanical properties due to unstable ion exchange and a deficiency of cell-adhesive signals prevent the maintenance of these good conditions for hepatocytes for a long period [42]. Thus, a hybrid alginate/galactosylated chitosan (ALG/GC) porous scaffold was fabricated by lyophilization, and the mechanical strength was enhanced by changing the ALG to GC ratio and controlling the freezing temperature [23,43]. Primary hepatocytes isolated from mouse seeded onto ALG/GC showed a 30% increase of hepatocyte attachment compared to the alginate scaffold alone. These results were due to good interactions between the ligands and receptors available in the appropriate combination of ALG and GC. In addition, hepatocyte functions such as albumin secretion and ammonia removal were significantly higher and were maintained for a longer time than on alginate scaffolds without chitosan.

Advances in polymer chemistry have aided the engineering of synthetic biomaterials to overcome the disadvantages of natural polymers. Poly(L-lactic acid) (PLLA), poly(lactic-co-glycolic acid) (PLGA) and poly(ε-caprolactone) (PCL) are among the wide variety of synthetic polymers available with the benefits of good mechanical properties, controllable degradation rates and easy accessibility. For example, collagen-coated PLGA scaffolds were to be useful for culturing rat hepatocytes, which exhibited urea synthesis after two weeks of culturing [44]. The culturing efficacy of PLLA 3D scaffolds was investigated by culturing porcine hepatocytes in the presence of hepatocyte growth factor (HGF). Liver specific functions were reported to be enhanced, with increased levels of albumin secretion, cytochrome P450, ammonia removal and urea synthesis compared to those of the control [45].

However, due to several issues, scaffold-based engineering of highly structured liver replacements is not really applicable. In some cases, the scaffold does not fully degrade and affecting amount of ECM deposited by cells, thus delaying the regeneration of neo tissue resembling natural liver and possibly causing fibrosis [32]. The inflammatory response also occurred with the biodegradation process in certain cases, even with non-toxic material [33,46]. Another significant drawback is a poor perfusion rate, which disturbs the smooth flow of nutrients and waste products, and hence affects the cell viability.

##### Microfluidic Platforms

The ability to work with small volumes of fluids flowing through micro channels with high analytical precision are the main advantages offered by microfluidic platforms for biomedical applications including tissue engineering [47–49]. A wide variety of microfluidic platforms have been developed for liver tissue engineering, and each addresses certain factors such as cell seeding method, cell density needed, gradient and flow rate of fluids and oxygen concentration, as well as differing in design and fabrication techniques [5].

Early work had fabricated a scaffold-based microbioreactor that allowed continuous perfusion of nutrients to the hepatocytes [34]. The scaffolds were designed to provide a 3D culture environment as well as mechanical support, while the microbioreactor consisted of chambers with individual channels to permit the flow of culture medium controlled by the low permeability of the filter system. More recently, with some improvements, Domansky *et al.* developed perfused multiwell plates where each well contained a scaffold-based bioreactor [26]. Hepatocytes were seeded onto each ECM-coated scaffold to deliver the optimum concentration of oxygen and biochemical force. Additionally, an external pneumatic diaphragm micropump was integrated to maintain a constant perfusion of culture medium, as well as model oxygen sensors to assess the oxygen tension received by hepatocytes. Another key feature of this multiple microbioreactor was that its design resembled conventional multiwell plates for tissue culture to enable ease of handling.

On the other hand, Goral *et al.*, cultured hepatocytes in 3D perfused microfluidic devices without the presence of biological or synthetic matrices [35]. The 3D microenvironment was maintained by a line of micropillars surrounding the cell culture chamber, and unlike other microfluidic devices, the base of the cell culture chamber also featured patterned micropillars to enhance cellular organization. The micropillars were designed to allow the continuous flow of culture media from two side microchannels and the bottom of the microstructure. The formation of gap junctions and extended bile canaliculi during *in vitro* hepatocyte culturing indicated that a 3D microenvironment could be induced in the absence of ECM provided with dense cell-cell interactions on a perfused microfluidic platform. More recently, another hepatocyte culturing technique utilizing gel-free microfluidic platforms was presented [36]. The authors proposed a multi-row square-pillar microstructure as the perfusion mechanism with a larger cell culture area. Up to 90% of the cultivated hepatocytes showed viability at day 5, supporting the hypothesis that the proposed design enables balance between the low shear stress and high mass-transfer rate experienced during cell seeding.

In general, many parameters have been considered in each development of microfluidic platforms for the use of liver tissue engineering; high cell density to favor cell-cell and cell-ECM interactions, proper cell seeding to reduce cell damage, a good perfusion rate to provide adequate nutrients as well as a sufficient oxygen supply to promote angiogenesis. However, because the liver comprises a microstructure of heterogeneously arranged cells, cell patterning technology for liver reconstruction could be compulsory. Thus, the high function and long term viability of engineered liver might be achieved with micropatterning technologies that can precisely position cells to closely mimic the natural pattern of the liver.

##### Micropatterning

Early work in the micropatterning of heterogeneous liver cells employed photolithography techniques requiring which cell-adhesive materials such as collagen, fibronectin and polylysine. Using photolithography, Bhatia *et al.* coated collagen on a substrate in specific regions to promote hepatocyte attachment. A second cell type, fibroblasts, were then seeded, these cells attached and occupied the remaining untreated areas with favor of serum-mediated attachment, thus forming a well-ordered pattern [37,50]. Regardless of the well-known photolithography technique, this approach of micropatterning is actually restricted when it is subjected to the efficiency of cell-cell and cell-substrate adhesiveness. For instance, the first cell type must strongly adhere to the patterned area and weakly adhere to the unpatterned area, and the opposite should be the case for the second cell type. Furthermore, the ordinary cell adhesion process is slow and uncontrollable, thereby decreasing the effectiveness of this technique for further application in liver tissue engineering.

Recent progress in surface engineering has introduced a controllable surface to dynamically regulate the interactions between cells and substrate for the successive patterning of heterogeneous cells. Cell sheets are a tissue engineering method utilizing a temperature-responsive cell culture dish. This special dish is prepared by covalently grafting a thin layer of polymer, poly(N-isopropylacrylamide) (PIPAAm), onto typical polystyrene cell culture dishes by electron beam radiation, which is sensitive to the culture temperature [38]. At a standard culture temperature of 37 °C, the PIPAAm-grafted surfaces behave as polystyrene dishes that enable cell adhesion, proliferation and culture. By decreasing the culture temperature to 32 °C, the polymer's lower critical solution temperature (LCST), the PIPAAm-grafted surfaces rapidly become hydrophilic, facilitating cultured cell detachment and forming cell sheets without any need of chemical or mechanical forces.

Hirose *et al.* have developed two cell co-culture arrangements of patterned primary hepatocytes and endothelial cells [51]. In the first arrangement, patterned co-culture was performed by utilizing an electron beam with a patterned mask to treat the PIPAAm-grafted surface. Hepatocytes were cultured under standard culture temperature and spontaneously detached below the LCST. Endothelial cells were then cultured on the same surfaces at 37 °C and occupied the exposed PIPAAm-grafted area to form heterogeneous cell patterns. In contrast, another arrangement included double layered co-culture achieved by covering the hepatocyte monolayers with endothelial cell sheets. Both arrangements appeared to maintain their differentiated state and functions for approximately one week and could be transferred with the desired shape. Maintaining the cell shape along with the ECM adhered onto the basal of cells sheet was another positive feature of this method. Compared to the conventional method using trypsin, this temperature-responsive cell culture dish offers a non-invasive harvesting method, as the cultured cells spontaneously detach in response to temperature change. Through examination by surface analysis and characterization, a group of proteins indicating the presence of ECM and good cell adhesion was found when endothelial cells were detached by simply reducing the temperature [52,53].

However, although cell sheet engineering is very efficient in cell detachment, this method is highly dependent on the surface chemistry of the thermo-responsive dish to preserve good cell adhesion in a micropatterned manner [24,54]. Numerous types of thermo-responsive dishes were developed to counteract several effects such as rapid dehydration due to poor grafting technique of PIPAAm on the surface. Furthermore, additional steps are required, such as microcontact printing of collagens to enhance the production of ECM because they are essential for stacking multiple types of cell sheets to form 3D heterogeneous tissue [55].

In contrast to the above-mentioned passive cell patterning, various approaches have been developed and are still under research to actively position cells in desired patterns. With the aid of external forces such as magnetism, optics, and electrokinetics, or by combining some of these, multiple cell types, such as hepatocytes and endothelial cells, can be precisely controlled and rapidly direct cell adhesion.

Ink-jet patterning, which uses a computer-aided design (CAD) system to position cells layer-by-layer for 3D organ building, offers rapid and high-resolution patterning of single and multiple cell types [39]. However, the sequential processes, including designing the organ using CAD, cell printing to form cell aggregates according to the CAD design and lastly, organ conditioning to promote cell maturation, are quite laborious and costly. Moreover, high concentrations of cells cannot be used because they may cause nozzle clogging during cell printing and thereby influence the patterning accuracy [56]. Recent advances in optical technology have led to laser-guided writing capable of simultaneously directing multiple types of cells via a laser beam [40]. However, the energy loading used is still a major concern, as it may lead to cell damage. Meanwhile, owing to the unique dielectric properties of every cell type, electrical force may have the ability to position multiple types of cells with high selectivity and accuracy. The electrical forces used for cell patterning at the micro scale involve electrophoresis [57] and dielectrophoresis [41,58], which are useful for transporting cells in microfluidic systems [59,60]. In addition, large numbers of cells can be patterned simultaneously without any need for cell pre-modification or labeling, signifying benefits for tissue engineering. In the next section, the fundamental principles of dielectrophoresis (DEP) will be briefly described along with a presentation of microelectrodes for liver cell patterning.

## 3. DEP for Liver Cell Patterning

### 3.1. Principles of DEP

DEP force is the movement of polarized particles within a medium when subjected to a non-uniform AC electrical field, as first described by Pohl in 1951. Biological cells as well as microbeads, DNA, protein, bacteria and so on, are good candidates for polarizable particles for manipulation by isolation, characterization, separation, and patterning [61–66]. Innovations in DEP research coupled with advanced microfabrication and microfluidic techniques have introduced methods for developing better tissue engineering tools, primarily for liver.

**Figure 3.**
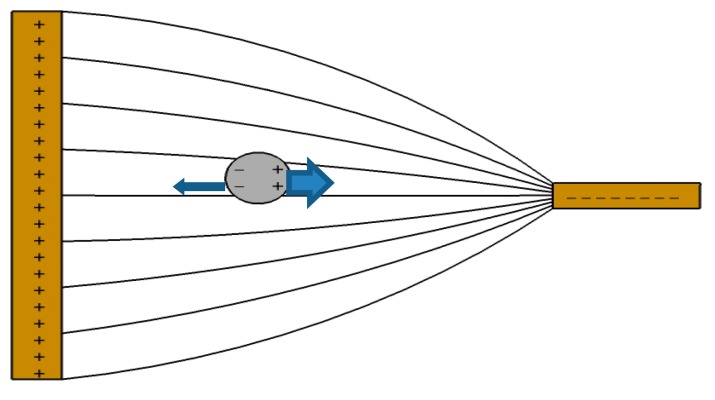
Principle of dielectrophoresis in an inhomogeneous electric field. Cells that are more polarizable than the surrounding medium are attracted towards the high electric field at the smaller electrode.

When suspended in a non-uniform AC electrical field, natural cells will become polarized and experienced DEP force, *F_DEP_*, given by Equation (1):

(1) FDEP=2πr3ε0εmRe[K(ω)]∇E2

where *r* is the radius of the cell; ε_0_ and ε_m_ are the permittivity of free space and the medium surrounding the cell, respectively; Re[K(ω)] is the Clausius-Mossotti factor; ∇ is the Del gradient operator; and E is the electrical field. The Clausius-Mossotti factor is further described by Equation (2):

(2) $$Re\left[K\left(\omega \right)\right]=\frac{\varepsilon_{p}^{*}-\varepsilon_{m}^{*}}{\varepsilon_{p}^{*}+2\varepsilon_{m}^{*}}$$

where *ω* is the angular frequency, and $\varepsilon_{p}^{*}$ and $\varepsilon_{m}^{*}$ are the complex permittivities of the cell and medium, respectively. Additionally:

(3) $$\varepsilon^{*}=\varepsilon -\frac{j\sigma }{\omega }$$

where *j* is the imaginary unit, *ε* is the permittivity, *σ* is the conductivity, and *ω* is the angular frequency of the given AC electric field. The direction of cellular movement is dependent upon the relative polarizability of the cell in the suspension medium according to the Clausius-Mossotti factor, which further depends on the applied frequency, *f* = *ω*/2*π*, and on the properties of both the suspension medium and the cells. As illustrated in Figure 3, in the case where the polarizability of cells is higher than that of the medium, the cells tend to move towards the smaller electrode, where the electrical gradient is high. If the cells are less polarizable than the medium, they will then move towards the low electrical gradient near the larger electrode. The attraction of the cells to high electric fields is identified as positive DEP and the repulsion from high electric fields is identified as negative DEP.

This phenomenon was believed to be beneficial for cell patterning. The fact that DEP is a frequency-dependent force enables cells to be precisely controlled and manipulated by a particular frequency and guided to form a desired pattern relative to the non-uniform electric field generated by the microelectrode.

### 3.2. Microelectrodes for Cell Patterning

Microelectrode devices designed specifically for cell patterning have particular requirements that differentiate them from other applications. Significant considerations in the development of microelectrode cell patterning include microelectrode geometry and dimensions for specific cell patterning and culture, as well as the patterning configuration used for DEP cell manipulation. Therefore, one must be aware of all the possible choices to ensure that the design is in line with the desired application. This section highlights the several types of geometry that exist, specifically configurations to control the patterning of liver cells.

#### 3.2.1. Microelectrode Geometry

In order to dielectrophoretically move the cells, a non-uniform electric field is necessary to generate unbalanced force on the suspended cells in the field. The non-uniform field can be created by applying voltage across geometrical electrodes [67], by placing an insulator between electrodes [68,69] or even electrodeless [70,71].

**Figure 4.**
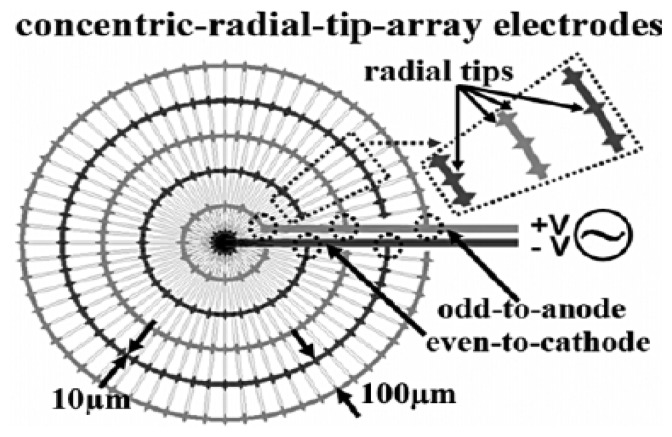
Concentric-stellate-tip microelectrode for 2D liver cell patterning. Reproduced with permission [74].

A wide range of microelectrode geometries have been demonstrated for patterning multiple types of cells with different formations [72]. The most common and cost-effective microfabrication technique, photolithography [67], can precisely form microelectrode gaps to create pearl-chain effects with the use of specific DEP configurations [73]. This approach was proven to provide significant effects in the patterning of complex and interconnected heterogeneous liver cells. Ho and colleagues, with their proposed microfluidic chip, have designed an array of concentric-stellate-tip microelectrodes to yield radial-patterned electric fields for dielectrophoretically manipulating viable liver cells [74]. As illustrated in Figure 4, the concentric-ring array electrodes were designed to generate radial electric fields with random pearl-chain effects.

To further form and align the hepatocyte cell chains with radial orientation, each ring included stellate tips to enhance the electric field gradient, thereby attracting the cells. The gaps between the adjacent rings were fixed at 100 μm to trap approximately 8 cells in a line. Such 2D concentric-stellate-tip microelectrodes were designed to purposely mimic the lobular structure of biological liver tissue, in which vascular endothelial cells were then trapped in between hepatocyte cell chains radiating outward from the center by DEP manipulation.

**Figure 5.**
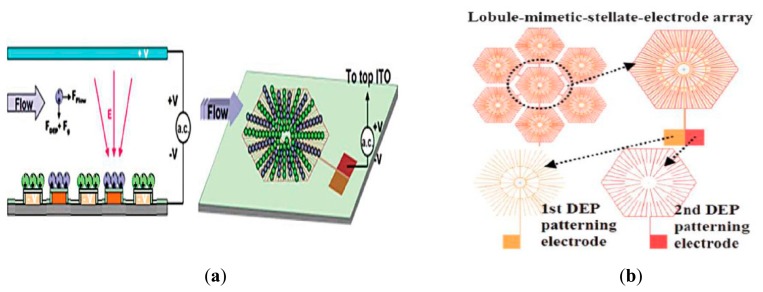
(**a**) Vertical setup for 3D heterogeneous cells patterning by DEP. (**b**) The lobule-mimetic-stellate-electrode arrays for 3D liver cell patterning. Reproduced with permission [28].

However, recently, Ho and other researchers have made some modifications in the microelectrode arrangement to achieve three-dimensional liver cell patterning by generating a vertical DEP force, as shown in Figure 5a [28]. In addition, they also made some changes to the liver-mimetic microelectrode design, which consists of two independent electrodes (Figure 5b). The first DEP patterning electrode functions to snare hepatocytes, while the second electrode is for snaring endothelial cells. Moreover, just as in the previous design, the outer part of both electrodes has a compact electrode design for denser cell patterning.

In another strategy, Schutte *et al.* utilized insulator-based DEP cell patterning to guide human primary hepatocytes and endothelial cells into a liver-sinusoid pattern [75]. Moreover, their microfluidic chip also provided continuous perfusion of culture media and automated cell seeding. A set of electrodes in the sidewalls of the microchannels with cross-sections in between was used to generate non-uniform electric fields to produce DEP force to guide hepatocytes surrounded by endothelial cells towards the assembly gaps in the well-designed cell culture chambers. By increasing the height of the gaps to 100 μm and decreasing the inclination angle to 30°, the numerical simulation results showed that the cells could experience high DEP forces to further assemble into the gaps in a liver sinusoid-like pattern within 2 minutes. Furthermore, the micropillars placed at the front and back of the assembly gaps were also found to successfully control the flow velocity and shear stress during cell seeding and culture. However, ongoing research for long-term sinusoid cell culture on-chip is still in progress; more parameters still need to be investigated, such as electrical effects on the cells and the essential culture conditions.

#### 3.2.2. Patterning Configuration

Live cells can maintain their viability under strong DEP forces under many conditions. Applying high-voltage electric pulses may cause cell damage such as electropermeabilization due to the high permeability of the cell membrane. Nevertheless, short-term exposure to certain ranges of frequency showed few acute changes in cells under DEP forces [76]. Furthermore, high-frequency electric fields can indirectly reduce electrochemical effects at the electrode, such as corrosion and bubble formation [77]. In terms of DEP manipulating buffer, a high-conductivity buffer is required to keep cells alive and to maintain their adherence capability. Media containing high concentrations of Ca^2+^ and Mg^2+^ ions are favorable for activating cadherins and integrins, molecules needed for cell adhesion [78]. However, a low-conductivity buffer is required for effective DEP cell manipulation. In addition to significantly reducing the heating effect, reducing the medium conductivity can minimize the electropermeabilization effect [79].

As described earlier, cells can be configured either by positive DEP (p-DEP) or negative DEP (n-DEP), depending on the working frequency. In the case of cell patterning, p-DEP is more appropriate because it attracts and assembles cells exposed to high electric gradient according to the microelectrode geometry. There were also some studies that utilized n-DEP for cell patterning. After an AC voltage was applied, n-DEP forces pushed the cells away from electrodes yet still reflected the electrode shape [80]. Moreover, an inverted design can also be used under n-DEP for patterning [81]. However, aside from the smaller forces generated by n-DEP, the main disadvantage for cell patterning is that the cells are unlikely to remain in the pattern design and rapidly move out of the design after removing the DEP forces.

Both horizontal and vertical p-DEP have been proven to successfully pattern hepatocytes and endothelial cells according to designed microelectrodes, closely mimicking the morphology of real liver tissue [28,74]. In early microelectrode designs, horizontal DEP forces were generated by two concentric electrodes placed on the same plane, identified as odd-ring and even-ring, which were subjected to an AC voltage. To achieve both robust DEP forces and high cell viability, the parameters were set to be below 5 V at 1 MHz with low-conductivity medium of 10 mS·m^−1^. Upon voltage application, in parallel, p-DEP forces guided the randomly distributed hepatocytes towards the high potential region provided by the stellate tips and the cells aggregated to it. The optimum cell density achieved then resulted in strong interactions between the cells due to the dipole induced by DEP and eventually formed a radial pearl-chain pattern from tip to tip [82]. To ensure high hepatocyte viability, the DEP manipulating buffer was replaced by standard cell culture medium without DEP presentation for some time. The pearl-chain formation allowed direct communication between cells, thus enhancing the adherent forces and preventing the well-patterned hepatocytes from falling apart. In addition, prior to the experiment, the substrate was coated with poly-D-lysine to facilitate the immobilization of the hepatocytes on the substrate and maintain the liver-like formation. Later, continuing the p-DEP manipulation, endothelial cells were loaded and resided in the empty spaces between the hepatocyte chains, mimicking the sinusoid pattern of vascular endothelial tissue in real liver. In another arrangement, Ho *et al.* utilized vertical p-DEP across two lower electrodes for trapping cells, and upper electrodes acted as ground electrodes biased with an AC voltage. The first electrode, as depicted in Figure 5b, was supplied with vertical p-DEP voltage to attract, trap and pattern hepatocytes according to the electrode design. Then, p-DEP forces were again used to move, snare and pattern endothelial cells according to the second electrode design, which sandwiched them between the first patterned hepatocytes to attain a heterogeneous cell pattern mimicking the morphology of biological tissue.

## 4. Applications

Liver transplantation therapy is limited by the shortage of organ donors and the need for continuous immunosuppression, which is costly and laborious. Thus, current progress in cell-based treatments, for example liver tissue engineering, offer great alternative methods of treatment by using cells instead of organs [83]. This much less invasive technique is also believed to be less immunogenic because the immunogenicity of allogeneic cells can be manipulated prior to implantation [84]. Furthermore, autologous cells also could be used because they are more immunologically compatible with the patient following *in vitro* adaptation.

Owing to the high regenerative capacity of hepatocytes, there could be additional advantages to culturing the cells in an *in vitro* system. However, it should be performed in an *in vivo*-like microenvironment and closely resemble the complex architecture of native liver tissue if the aim is to replace the numerous *in vivo* liver functions. Hence, DEP-based cell patterning technology is among the best techniques for precisely patterning heterogeneous liver cells to mimic the morphology of biological liver to maintain hepatocyte functionality, coupled with microfluidic systems to provide an *in vivo*-like culturing microenvironment for high cell viability.

An *in vitro* hepatocyte-based cell culture model for drug screening is gaining interest in the pharmaceutical field because conventional animal testing methods are costly and there are an increasing number of ethical issues [85,86]. Hence, efficient, reliable, precise and cost-effective tools for liver toxicity analysis are in high demand. Recently, there have been several reviews introducing lab-on-a-chip devices incorporating liver cells with various features to maintain their phenotypic function for drug studies [87,88]. For example, Toh *et al.* developed a microfluidic hepatocyte chip called the 3D HepaTox Chip, comprising eight parallel cell culture channels that were individually subjected to outputs of a concentration gradient generator [25]. Five hepatotoxic models of multiple doses were demonstrated to determine the median lethal concentration values (LC_50_) and were found to be correlated with the *in vivo* values. However, inconsistent results appeared after 72 h of cell culture. This might be due to the absence of endothelial cells and the inability to isolate dead cells from viable cells, hence slowly lost their function.

In contrast, DEP-based liver cell micropatterning is capable of assembling hepatocytes and endothelial cells into liver-like sinusoid patterns [75]. The high selectivity of viable cells via DEP and the other advantages offered by microfluidic systems proved to be significant in developing an *in vitro* liver model for high-throughput drug screening. In other advanced studies, more *in vitro* organ models such as liver, intestine, lung and other target tissues were combined into one microfluidic platform separated by chambers for a total drug screening system [89–91].

## 5. Conclusions and Outlook

Comprehensive reviews on the theory, microelectrode design and applications of dielectrophoresis (DEP) have been thoroughly described by experts [92,93]. The integration of DEP into microfluidic platforms has gained a great deal of interest for the *in vitro* engineering of complex tissue organization such as that of liver. The complexity of biological liver often refers to the heterogeneous cells that are uniquely structured to perform their physiological roles. Thus, patterned co-culture of parenchymal hepatocytes and other non-parenchymal cells such as endothelial cells and fibroblasts is crucial in the construction of engineered liver tissue to obtain maximum cell-cell and cell-ECM interactions.

In competition with other passive and active micropatterning techniques, DEP is a key technique for spatially patterning heterogeneous liver cells and has substantial benefits including rapid in-parallel patterning, high accuracy, no need for prior cell modification, simple and low-cost equipment as well as the ability to integrate with other techniques. However, the progress of DEP-based liver cell patterning is still preliminary, and there are many complications that need improvement. Further studies toward the development of 3D liver cell patterning are necessary because creating 3D effects by vertical DEP setup is limited by several factors such as gravity and cell migration. Thus, the layer-by-layer assembly approach offered by cell sheet technology might be compatible with DEP-based techniques for 3D liver tissue construction. Furthermore, the combination of a promising hydrogel, gelatin methacrylate (GelMA), with a DEP technique for 3D microscale organization has recently been reported [94]. In another approach, two well-known micropatterning approaches, optical tweezers and DEP, were successfully integrated to form optoelectronic tweezers that could dynamically manipulate and pattern liver cells (HepG2) via negative DEP force using a light-driven optoelectronic DEP chip [95]. Yang *et al.* in a recent approach also utilized HepG2 cells featured in their TiOPc-based optoelectronic DEP chip. The laser diffraction-induced dielectrophoresis with optimum optical and electrical parameters setting for the cell patterning have showed similar growth rate and morphology to those cultured on normal cell culture dish [96]. In general, with the vast progress in the field of liver tissue engineering, there is a great deal of potential for working towards whole-organ implants as well as for drug discovery studies.

## Figures and Tables

**Table 1.** Comparison between available engineering approaches.

Engineering Approaches	Features/Tools	Fabrication Technique	Advantages	Limitations	References
**Scaffold-based**	Natural biomaterials Synthetic polymer Hydrogels	Needs an expert to handle	3-D environment No external forces	Not applicable for complicated structure tissue Need highly-control over microscale histoarchitecture (*i.e.*, pore size, biodegradability, biocompatibility) Poor mass transport properties Inflammatory response Weak real-time imaging system	[29–33]

**Microfluidic Platforms**	Scaffold-based microbioreactor Microchannels perfusion Micropillars perfusion Microwell/microplate arrays	Low cost Easy to handle	3-D environment Multicellular culture system Sophisticated control of a dynamic environment Integrated microdevices Real-time imaging system Point-of-care device	Needs special attention to surface chemistry of substrate	[5,26,34–36]

**Micropatterning**	Photolithography Switchable surface - Cell sheets engineering Magnetism Optics – optoelectronic DEP Electrokinetics – Dielectrophoresis (DEP)	Low cost Easy to handle	2-D and 3-D environment 3-D patterned cell culture system Multicellular culture system Integrated microdevices Real-time imaging system Point-of-care device	Needs special attention to surface chemistry of substrate	[37–41]
